# Detecting COVID-19 in chest images based on deep transfer learning and machine learning algorithms

**DOI:** 10.1186/s43055-021-00524-y

**Published:** 2021-06-11

**Authors:** Seyed Masoud Rezaeijo, Mohammadreza Ghorvei, Razzagh Abedi-Firouzjah, Hesam Mojtahedi, Hossein Entezari Zarch

**Affiliations:** 1 Department of Medical Physics, Faculty of Medical Sciences, Tarbiat Modares University, Tehran, Iran; 2 Department of Electrical and Computer Engineering, Tarbiat Modares University, Al-Ahmad and Chamran Cross, Tehran, Iran; 3 Cellular and Molecular Research Center, Yasuj University of Medical Sciences, Yasuj, Iran; 4 School of Electrical and Computer Engineering, University of Tehran, Tehran, Iran

**Keywords:** COVID-19, Chest CT images, Deep transfer learning, Machine learning

## Abstract

**Background:**

This study aimed to propose an automatic prediction of COVID-19 disease using chest CT images based on deep transfer learning models and machine learning (ML) algorithms.

**Results:**

The dataset consisted of 5480 samples in two classes, including 2740 CT chest images of patients with confirmed COVID-19 and 2740 images of suspected cases was assessed. The DenseNet201 model has obtained the highest training with an accuracy of 100%. In combining pre-trained models with ML algorithms, the DenseNet201 model and KNN algorithm have received the best performance with an accuracy of 100%. Created map by t-SNE in the DenseNet201 model showed not any points clustered with the wrong class.

**Conclusions:**

The mentioned models can be used in remote places, in low- and middle-income countries, and laboratory equipment with limited resources to overcome a shortage of radiologists.

## Background

Infectious pathogenic microorganisms, such as viruses, cause diseases. These diseases are one of the critical agents that threaten human health, for they are deadly acute diseases and infectious and can be spread from one person to another [1]. The spread of coronavirus (COVID-19) has been a great global concern because of threatening the people’s health [2], and there is no effective treatment to cure the disease [3]. In December 2019, pandemic COVID-19 appeared in Wuhan, China. Severe acute respiratory syndrome coronavirus 2 (SARS-CoV-2) caused the COVID-19 pandemic disease [4]. After the incubation period of about 2-14 days, the clinical presentation of COVID-19 begins, including fever, cough, and shortness of breath [5]. Because of the incubation period, COVID-19 can be spread even by asymptomatic persons. The World Health Organization (WHO) has suggested physical distancing and contact tracing in controlling the spread of COVID-19 [6]. An essential step in this process is the efficient and accurate detection of the COVID-19 patients, in which due to prevent spreading the virus, patients receive rapid treatment and become isolated. Various tests for diagnosing COVID-19 disease are available. These tests include reverse transcription-polymerase chain reaction (RT-PCR), loop-mediated isothermal amplification (LAMP), lateral flow assays (LFAs), enzyme-linked immunosorbent assay (ELISA), and computed tomography (CT) scan [7]. RT–PCR is a gold standard test and one of the most widely used laboratory techniques to detect the COVID-19 [8, 9]. However, screening every person affected by the virus in developing countries, with lack of laboratory equipment, is challenging. Furthermore, taking tests longs a few hours to a few days and it is time-consuming, and error-prone in the current emergency. Moreover, RT-PCR has a low sensitivity, and false-positive results have been reported [10]. Therefore, to prevent and to control COVID-19, a faster and reliable detecting modality is recommended. CT images are widely used for COVID-19 screening less developed countries, where an available number of test kits are low. Some studies have shown that a chest CT scan helps physicians assess and optimize prevention and control measures [11–13]. Therefore, the screening of CT images can be used as an alternative to laboratory tests. However, there is a limited number of radiologists in every hospital to interpret CT images. Therefore, an accurate and fast method is required to overcome this problem. Moreover, CT images provide quantitative information, but only qualitative information is reported due to the lack of computerized tools to process [14]. Image processing is a technique to extract useful information from an image. Recently, the deep learning model is preferred for quantitative image analysis [15, 16]. Deep learning diagnoses the disease and prepares suitable prediction models to assist doctors in developing effective treatment plans. Therefore, the automatic and quantitative analysis of CT images can be done through deep learning-based approaches [17–19]. One of these approaches is transfer learning, which is a sub-branch of deep learning. Transfer learning improves learning in a new task through the transfer of knowledge from a related task that has already been learned [20–22].

Machine learning (ML) and deep transfer learning methods increase the ability of researchers to sense how to analyze the common variations which will lead to disease [23]. These methods comprise conventional algorithms such as support vector machines (SVMs), decision tree (DT), random forest (RF), logistic regression (LGR), and k-nearest neighbors (KNN) [24], and deep learning algorithms like convolutional neural networks (CNNs). The SVM is a classification method that transforms a training dataset to a higher dimension. To separate the two classes with minimum classification errors, it optimizes a hyperplane [25]. The DT creates a tree-structured model to define the relationships between features and a class label [26]. The RF is a DT ensemble algorithm that through a re-sampling process called bootstrap aggregation creates multiple trees [27]. LGR models are the probability of data points belonging to a particular class according to independent features’ value. It then uses this model for predicting that a given data point belongs to a particular class [28]. The KNN is a classifier that trains by comparing a certain unlabeled data point with the training dataset [29]. CNN has shared weights and replicated filters on each layer with local connectivity without manual feature extraction. There are two types of layers, including feature extractors and trainable classifier [30]. There are different types of CNN architecture, including ResNet, DenseNet, VGGNet, InceptionV3, MobileNet, and EfficientNet [31]. The employed models’ core structure is explained in the “CNNs and proposed deep transfer learning models” subsection.

Although the RT–PCR test is the gold standard for screening suspected cases of COVID-19, this test is time-consuming and has false-positive results and insufficient sensitivity. Therefore, an automated method for diagnosing COVID-19 in chest CT images is required. The automatic analysis of CT images with CNN models has started to get further interest. These analyses can be done through deep transfer learning and ML methods so that they can accelerate the analysis time. In the deep transfer learning method, networks’ weights can train on large datasets and apply fine-tuning of the pre-trained models on small datasets. As it comes to our knowledge from the literature review, there are no any records for investigating extensively deep transfer learning and ML methods to recognize infected COVID-19 patients by chest CT images. Thus, in this study, the inductive transfer learning for the pre-trained CNN models, DenseNet201, ResNet50, VGG16, and Xception, was used to differentiate COVID-19 patients suspected. These models are considered among the most popular pre-trained CNN architectures used in the literature based on the recent survey by Khan et al. [32]. We aimed to investigate these classifiers to gain the maximum feasible accuracy on the COVID-19 diagnosis task independently from the chosen CNN architecture. We used the pre-trained weights on the ImageNet dataset as a start point for all models. Training this dataset helps the model to better general apparent patterns that are in image data. Using pre-trained weights on ImageNet for training small datasets helps the model to converge faster and easier.

The current study was conducted in two sections. In the first section, the output of pre-trained models was applied to differentiate COVID-19 patients from suspected. In the second section, ML methods, including RF, SVM, DT, KNN, and LGR, were used to classify patients. In this manner, the pre-trained methods’ output without any feature selection was applied as the input to the ML algorithms. The combination of different pre-trained models with ML algorithms was compared with the classification deep transfer learning models’ performance. Hence, extensive comparative analyses were performed to evaluate the models’ performance using various performance metrics such as accuracy, recall, precision, and f1-score statistics. This study briefly aimed to have proposed an automatic prediction of COVID-19 disease using chest CT images based on deep transfer learning models and ML algorithms.

## Methods

### Data set and data acquisition

**Fig. 1:**
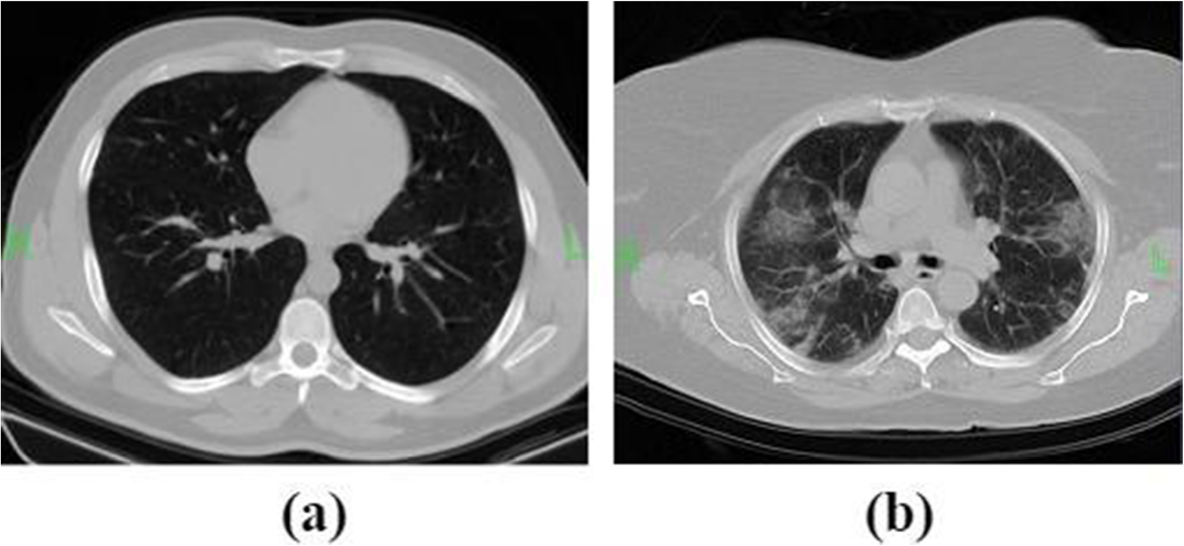
Sample of CT images from patients with suspected COVID-19 (**a**) and confirmed (**b**)

**Fig. 2:**
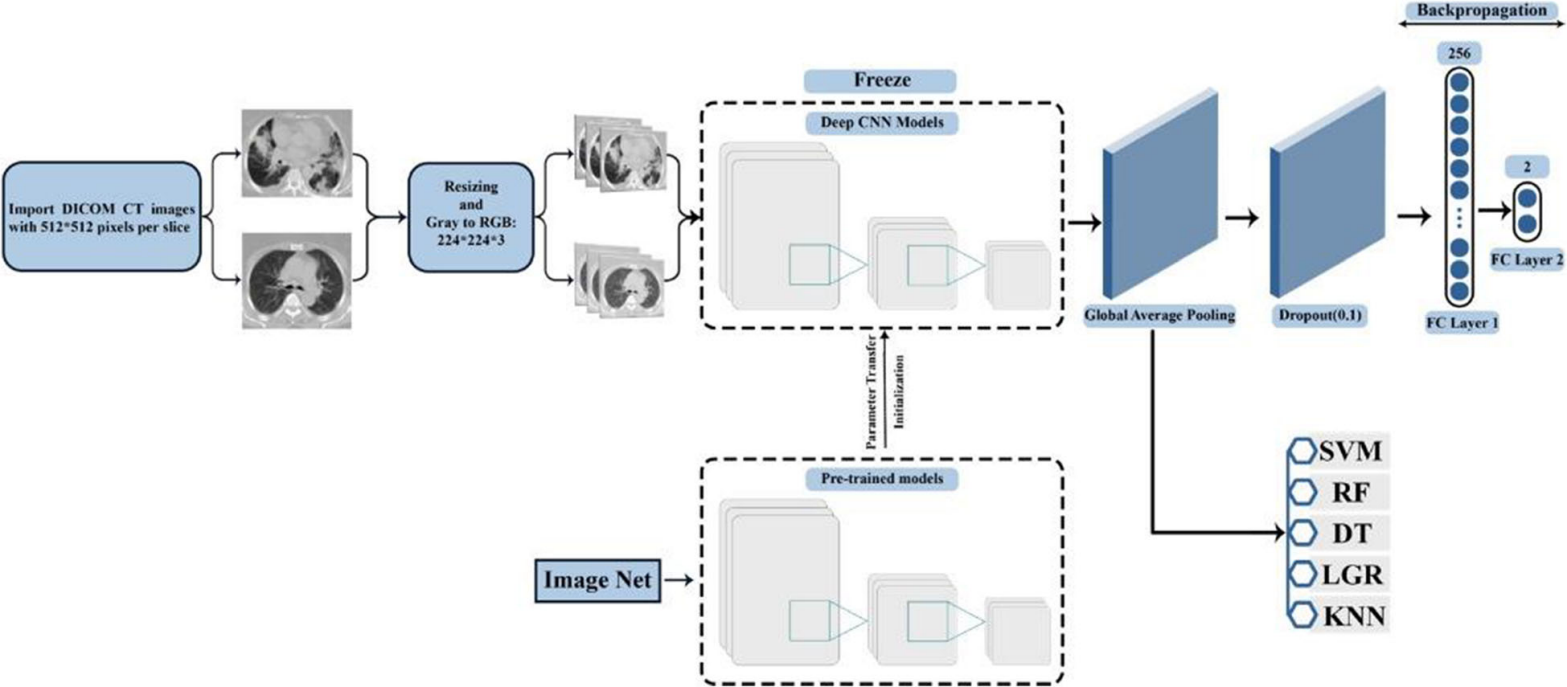
Graphical abstract

The dataset consisted of 5480 samples in two classes, including 2740 CT chest images of patients with confirmed COVID-19 and 2740 images of suspected cases. In the experimental analysis, 4400 images of the dataset were used as training data, and 1080 images as test data. It is necessary to mention that slices of each person were not divided between both training and test sets. The current study was carried out between 28 April 2020, and 3 September 2020. To manage COVID-19, all patients with a rapid respiratory rate over 30 per minute, fever over 37.8 °C, hypoxemia, dyspnea, cardiovascular disease, hypertension, diabetes mellitus, underlying pulmonary diseases, and immunodeficiency underwent non-contrast chest CT examinations. In our center, all patients must perform the PCR test and CT imaging to clarify COVID-19. A physician for screening and diagnosing COVID-19 reviewed medical records and imaging. All patients, both clinical findings and chest CT findings compatible with COVID-19 pneumonia, were located in the confirmed COVID-19 group. CT scans and laboratory tests confirmed that some patients had other lung infections. These patients had some common symptoms with confirmed COVID-19 patients. In these patients, CT imaging’s initial diagnosis was difficult, so additional laboratory tests were performed. That is why we named them suspected COVID-19. Non-contrast CT chest examinations were performed with a 16-slice CT scanner (Somatom Emotion; Siemens Medical Solutions, Forchheim, Germany) with the protocol as follows: kVp = 110, mAs = 90, slice thickness = 2 mm, matrix size = 512 × 512, voxel size = 0.714 mm, 0.714 mm, 2 mm. In Fig. 1, chest CT images of patients with suspected COVID-19 and confirmed are represented. The graphical abstract of the study is displayed in Fig. 2.

### CNNs and proposed deep transfer learning models

CNN is a class of deep learning models of data processing and analysis, which is an inspired design by the structure of the human visual cortex [33]. CNN is designed to learn spatial hierarchies of features through a backpropagation algorithm, from low- to high-level patterns. The CNN typical architecture includes repetitions of a stack of multiple convolution layers and pooling layers followed by one or more fully connected layers [34]. The convolution layer is an essential layer of the CNN model composed of several convolution kernels based on moving the input image with the selected filter to extract different feature maps. The size and number of kernels are two key hyperparameters that define the convolution operation. The size is typically 3 × 3, but sometimes are 5 × 5 or 7 × 7. The number of kernels is arbitrary and specifies the depth of output feature maps. In general, in the convolution layer, each of the output feature maps can be combined with more than one input feature map as follows:

1 $$ {x}_j^l=f\left(\sum \limits_{i\text{\EUR} Mj}{x}_j^{l-1}\ast {k}_{ij}^l+{b}_j^l\right) $$

Where the output of the current layer is ${x}_j^l$, ${x}_j^{l-1}$ is the previous layer output, ${k}_{ij}^l$ is the kernel for the present layer, and ${b}_j^l$ are the biases for the current layer. *M*_*j*_ represents a selection of input maps. The outputs of convolution are then passed per a nonlinear activation function. Rectified linear unit (ReLU) is the most common nonlinear activation utilized as an activation function [35]. It can be defined as:

2 $$ \mathrm{F}\ \left(\mathrm{x}\right)=\max\ \left(0,\mathrm{x}\right) $$

ReLU does by thresholding values at 0. When x < 0, it outputs 0, and conversely, when x ≥ 0, it outputs a linear function.

A pooling layer enables a specific down-sampling action, which reduces the feature maps dimension, the number of subsequent learnable parameters, and costs. It is necessary to mention that in any of the pooling layers, there is no learnable parameter. Therefore, hyperparameters in pooling operations are similar to convolution operations. The most common type of pooling operation is max pooling, which extracts the maximum value in the input maps, and discards all the other values. The global average pooling is another pooling operation. In this pooling, a powerful method of downsampling is performed with retaining the depth of feature maps. A feature map is downsampled into a 1 × 1 array using the average of all the elements. The global average pooling is applied before the fully connected layers [36]. Pooling operation can be formulated as:

3 $$ {x}_j^l= down\left({x}_j^{l-1}\right) $$

Where down (.) represents a sub-sampling function.

The output feature maps of the final convolution layer are typically transformed into a single vector, and the neurons are connected to all the activation functions from the previous layer. Each convolutional layer has a filter (*m*1). The output ${\mathrm{Y}}_{\mathrm{i}}^{\mathrm{l}}$of layer l consists of ${m}_1^l$ feature map of with size ${m}_2^l$ ×${m}_3^l$. The *i*th feature map, ${\mathrm{Y}}_{\mathrm{i}}^{\mathrm{l}},$ is calculated on the bases of Eq. 4:

4 $$ {Y}_i^{(1)}=f\left({B}_i^{(l)}+\sum \limits_{j=1}^{m_i^{\left(l-1\right)}}{k}_{i,j}^{(1)}\times {Y}_j^{\left(l-1\right)}\right) $$

Where ${B}_i^l$ demonstrates the bias matrix and ${K}_{i,j}^l$ the filter size.

The processing phases of the fully connected layer are shown in Eq. 5, if (*l* −1) is a fully connected layer;

5 $$ {Y}_i^{(1)}=f\left({Z}_i^{(l)}\right)\  with\ {Z}_i^{(l)}=\sum \limits_{j=1}^{m_i^{\left(l-1\right)}}{w}_{i,j}^{(1)}\times {Y}_j^{\left(l-1\right)}\Big) $$

Based on each task, an appropriate activation function needs to be selected. A softmax function is an activation function applied to the multiclass classification and the values in two classes of “0” and “1” interpreted [37].

The DenseNet201, ResNet50, VGG16, and Xception models are considered and described briefly in this section [30]. DenseNet201 includes densely connected CNN layers. In a dense block, the outputs of each layer are associated with all successor layers. Put merely, DenseNet201 organized with dense connectivity between the layers. The features extracted from the DenseNet201model is a 1920-dimensional space. ResNet50 is a usual feedforward network with a residual connection containing 50 layers, 49 convolution layers, and one fully connected layer. The features extracted from the ResNet50 model is a 2048-dimensional space. The image’s input size is usually set to 224 × 224 pixels, and the size of the filter can be selected to 3 ×3 or 5 ×5 pixels. The VGG16 architecture includes two convolutional layers such that both use the ReLU activation function. Followed, a single max-pooling layer and several fully connected layers also use a ReLU activation function. In this model, the convolution filter size is 3 × 3 filters with a stride of 2. The features extracted from the VGG16 model is a 512-dimensional space. Xception or Extreme Inception is a linear stack of depth wise detachable convolution layers with residual connections. In this model, except for the first and last modules, the 36 convolutional layers are structured into 14 modules. This architecture does not evaluate spatial and depth-wise correlations simultaneously and deals with them independently. The features extracted from the Xception model are a 2048-dimensional space.

### Machine learning methods

RF is a meta-learner that works by building many numbers of decision trees during the training process. The RF method only needs to determine two parameters for creating a prediction model, including the number of classification trees desired and prediction variables. Simply put, to classify a dataset, a fixed number of random predictive variables is used, and each of the samples of the dataset is classified by several trees defined [38]. SVM is a method to make a decision border between two classes that predicts labels using one or more feature vectors. The mentioned decision boundary is known as the hyperplane, with a maximum margin separating negative and positive data [39]. The output of an SVM classifier is given in Eq. 6, wherein w and x are the normal vectors to the hyperplane and the input vector, respectively.

6 $$ \mathrm{u}=\overrightarrow{w}.\mathrm{x}-\mathrm{b} $$

Maximizing margins can be determined as an optimization subject: minimize Eq. 7 concerning Eq. 8, where xi is ith training sample, and yi is the correct output of the SVM model for ith training.

7 $$ \frac{1}{2}{\left\Vert \overrightarrow{w}\right\Vert}^2 $$

8 $$ {y}_i\left(\overrightarrow{w}.\overrightarrow{xi}-b\right)\ge 1,\forall i $$

DT algorithm is a data mining induction method that recursively divisions a data set of records using the greedy method until all the data items belong to a specific class. The structure of this model is created of a root, internal, and leaf nodes. To classify new data records, the tree structure is used. At any internal node of the tree, making decisions about the best split is made by using impurity measures [40]. KNN classifier is a nonparametric classifier that provides good performance for optimal values of k. In the KNN rule, a test sample belongs to the class mostly represented among the k-nearest training samples, and classification is performed by calculating the distance between the selected features and the k-nearest neighbors [29]. The Euclidian distance to determine the spaces among the features can be calculated as follows: If two vectors x_i_ and x_j_ are given, the difference between x_i_ and x_j_ is:

9 $$ D\ \left({x}_{\mathrm{i}},{x}_j\right)=\sqrt{\sum_k^{n=1}{\left(\left( xik- xjk\right)\right)}^2} $$

LGR model is used when the value of the target variable is categorical, or is either a 0 or 1. A threshold is usually determined that demonstrated what value they will be put into one class vs. the other class [28]. The logistic regression model as follows:
10

### Experimental setup

The inductive transfer learning for the pre-trained CNN models, which are DenseNet201, ResNet50, VGG16, and Xception, was used to differentiate COVID-19 patients from suspected. In the inductive transfer learning method, the target duty is different from the source duty, no matter when the target and source domains are the same or not. Therefore, for inducing an objective predictive model fT (.) for use in the target domain, some labeled data in the target domain are needed. Based on “What to transfer,” there are different approaches to transfer learning that we used parameter transfer. Parameter transfer assumes that the model’s hyperparameters, the source, and target tasks share some parameters or prior distributions. Therefore, by finding the shared parameters or priors, knowledge can be transferred through tasks. This study was conducted in two sections. In the first section, the output of pre-trained models was used to differentiate patients with confirmed COVID-19 from suspected cases. Before training, we resized all the images into 224-pixel width and 224-pixel height in 3 channels for faster processing. The used structure for the four models was the same: the last convolutional block + model. Output + GlobalAveragePooling2D + Dropout (0.1) + Dense (256, activation= “ReLU”) + Dense (2, activation= “softmax”). It should be noted that only the last four layers were trained, and the rest of the pre-trained model layers were frozen. Finally, the performance of these models was obtained using four criteria as follows:

11 $$ \mathrm{Accuracy}=\left(\mathrm{TN}+\mathrm{TP}\right)/\left(\mathrm{TN}+\mathrm{TP}+\mathrm{FN}+\mathrm{FP}\right) $$

12 $$ \mathrm{Recall}=\mathrm{TP}/\left(\mathrm{TP}+\mathrm{FN}\right) $$

13 $$ \mathrm{Precision}=\mathrm{TP}/\left(\mathrm{TP}+\mathrm{FP}\right) $$

14 $$ \mathrm{F}1-\mathrm{Score}=2\times \left(\mathrm{Precision}\times \mathrm{Recall}\right)/\left(\mathrm{Precision}+\mathrm{Recall}\right) $$

TP, FP, TN, and FN represent the number of true positive, false positive, true negative, and false negative, respectively. We used the dimensionality reduction method “t-distributed stochastic neighbor embedding (t-SNE)” to visualize high-dimensional data by giving each data point in a two-dimensional map [41]. Therefore, t-SNE aims to preserve the significant structure of the high-dimensional data so that, put merely can be displayed in a scatterplot. t-SNE using a gradient descent method minimizes a Kullback-Leibler divergence between a joint probability distribution in the high-dimensional space and a joint probability distribution in the low dimensional. The pairwise similarities in the high-dimensional original data map as follows:
15

With conditional probabilities:
16

T-SNE has a tunable parameter, “perplexity,” which declares how to balance regard between local and global aspects of data. The perplexity is a guess of the number of close neighbors at each point. The perplexity value has a complex effect on the resulting image, and its value tuned to 200 for presented t-SNE in our study. We drew t-SNE plots for six different situations which including original CT images, Conv2-layer10, Conv15-layer56, GlobalAveragePooling layer, FC layer-layer 1, and FC layer-layer 2.

In the second section, we used ML methods, which include RF, SVM, DT, KNN, and LGR, to classify patients. In this manner, we entered the output of pre-trained methods into ML algorithms and performed the ML algorithms classification. The structure used to do this is as follows: the last convolutional block + model. Output + GlobalAveragePooling2D + predict datasets+ ML algorithms. The performance metrics of ML models were obtained similarly to pre-trained models. The performance metrics of ML models was obtained as the same as the pre-trained models.

All experiments, including data preprocessing and analysis, were performed on the Google Cloud computing service “Google Colab” (colab.research.google.com) using programming language Python and framework Tensor Flow. We used the following parameters to compile pre-trained models: optimizer= “Adam,” loss= “Categorical Crossentropy.” For all experiments, the batch size, learning rate, and the number of epochs were experimentally set to 64, 0.001, and 100, respectively.

## Results

We used chest CT images for screening and diagnosing COVID-19 disease. Popular pre-trained models such as DenseNet201, ResNet50, VGG16, and Xception and the combination of these pre-trained models with ML algorithms, including RF, SVM, DT, KNN, and LGR, have been trained and tested on chest CT images.

### Deep transfer learning models analysis

**Fig. 3:**
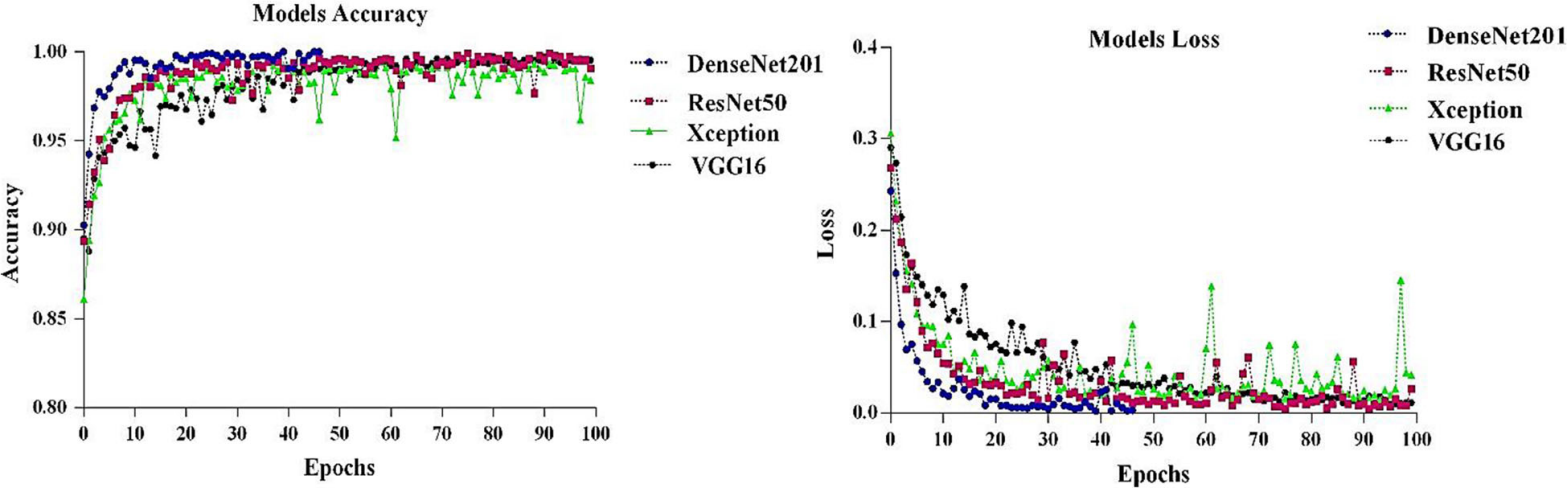
The values of accuracy and loss for the pre-trained models

The values of accuracy and loss for the pre-trained models are given in Fig. 3. For all pre-trained models, the training step has been carried out to the 100 epochs. Also, a similar early stopping mechanism to the training process was applied for all models so that if accuracy and validation accuracy reached the value of one, the entire learning was stopped. As shown in Fig. 3, for the DenseNet201 model, the learning was stopped at the 47th epoch by the early stopping criteria. It can be seen that the highest training accuracy was obtained with the DenseNet201 model and then have other models show a fast-training process. ResNet50, Xception, and VGG16 models have almost the same function. In four pre-trained models during the training step, loss values decrease. As shown, the DenseNet201 model decreases loss values faster than other models.

**Fig. 4:**
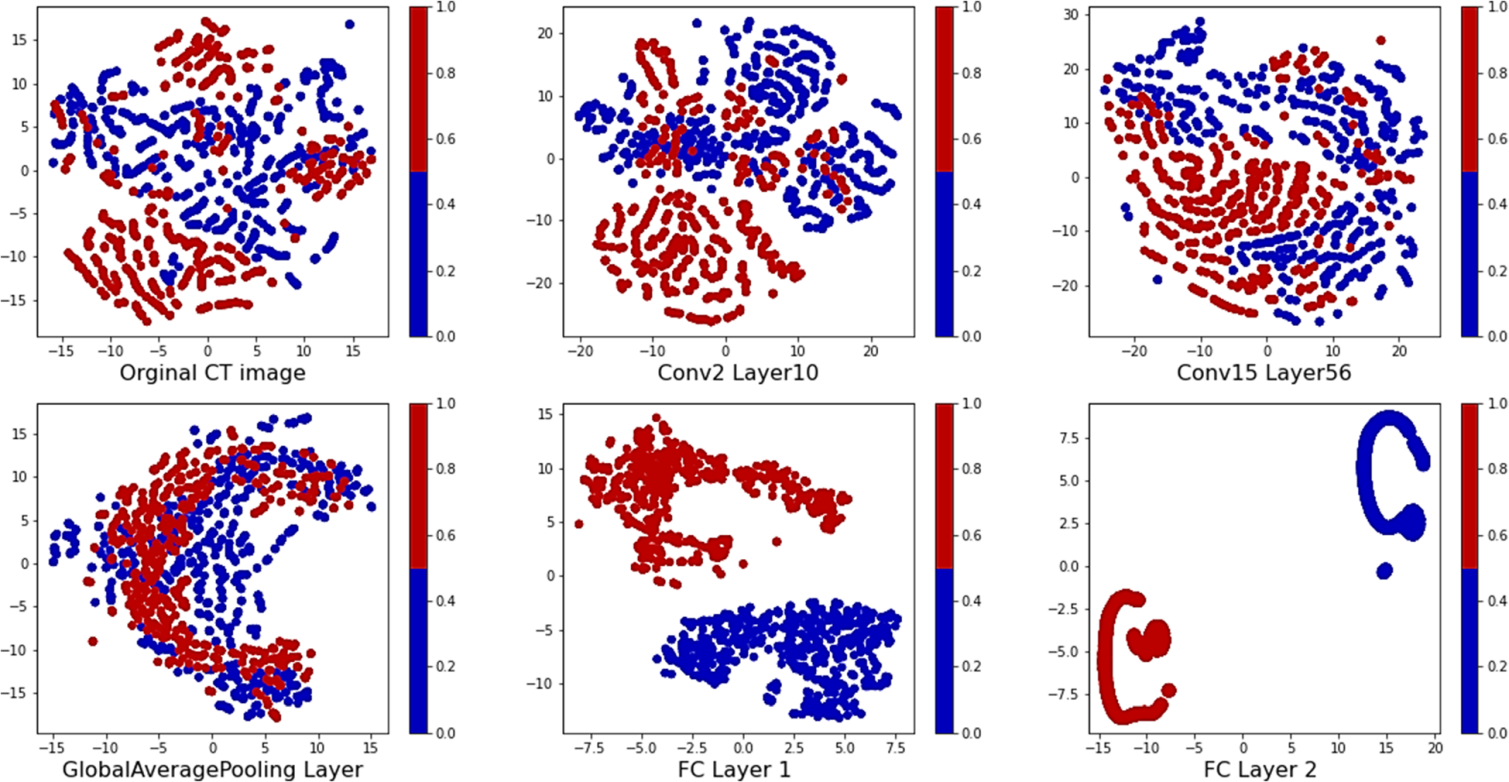
Data visualizations with the t-SNE method for the DenseNet201 model

To visualize data in a two-dimensional map, we used the dimensionality reduction method t-SNE, that data was displayed in a scatterplot. We drew t-SNE plots for six different situations: original CT images, Conv2-layer10, Conv15-layer52, GlobalAveragePooling layer, FC layer-layer 1, and FC layer-layer 3. Figure 4 shows the created map by t-SNE in the DenseNet201 model, and in this model, not any points were clustered with the wrong class. These results reveal the strong performance of the t-SNE method.

**Fig. 5:**
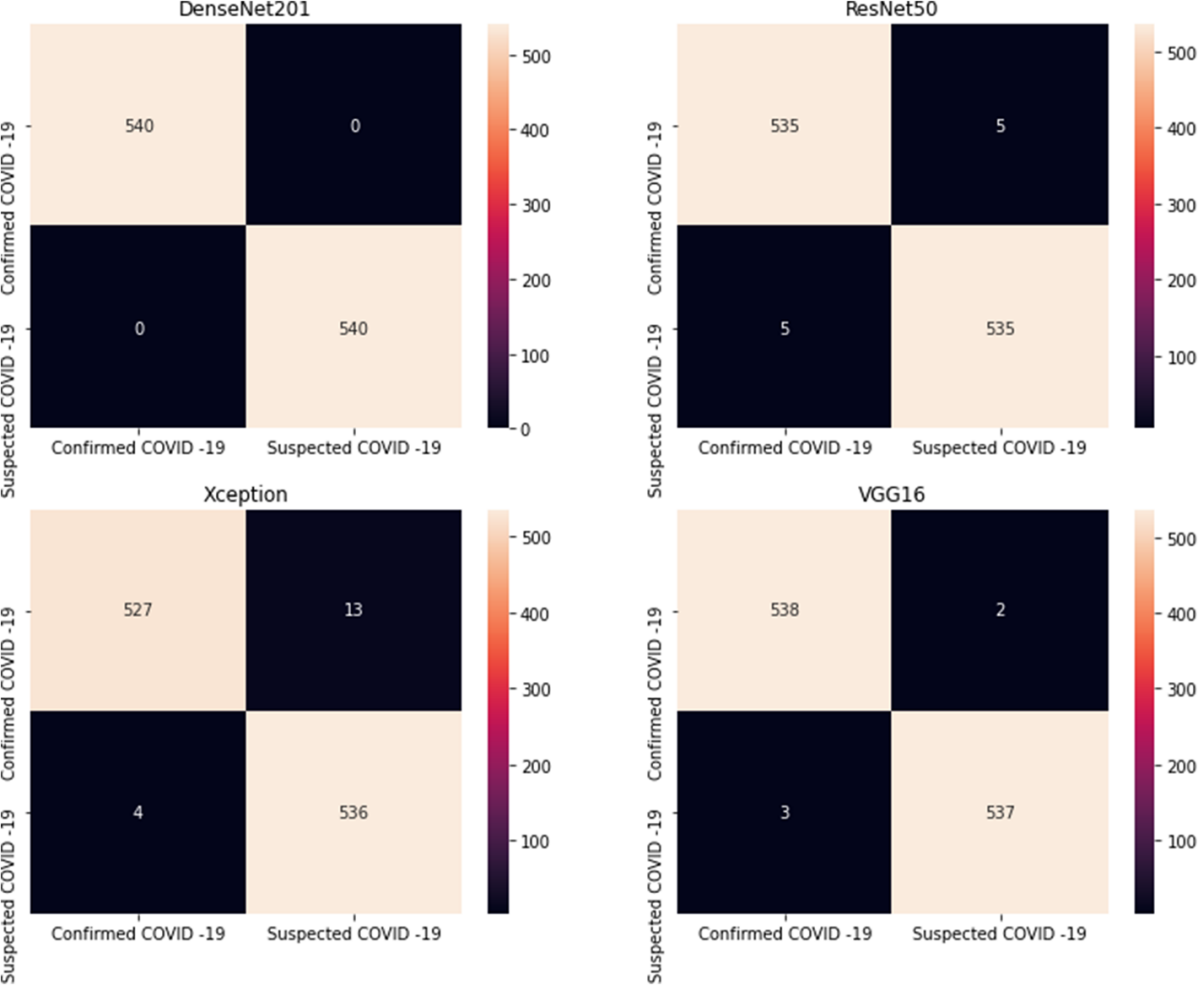
Confusion matrix analyses and ROC plots of the pre-trained models

In another detailed review, comparing four pre-trained models using the test data are presented in Table 1, and Fig. 5. As shown, the DenseNet201 model has obtained the highest training as the accuracy of 100%. Furthermore, we received the best performance as a recall of 100%, Precision 100%, and f1-score value of 100% for the DenseNet201 pre-trained model. However, the lowest performance values were yielded,98.42%, for parameters the accuracy, recall, precision, and f1-score value for the Xception pre-trained model.

**Table 1:** The values of accuracy, recall, precision, and f1-score were obtained for the pre-trained models

Model	Performance metrics (%)			
	Accuracy	Recall	Precision	F1 score
DenseNet201	100	100	100	100
ResNet50	99.1	99.1	99.1	99.1
VGG16	99.53	99.53	99.54	99.53
Xception	98.42	98.42	98.43	98.42

**Fig. 6:**
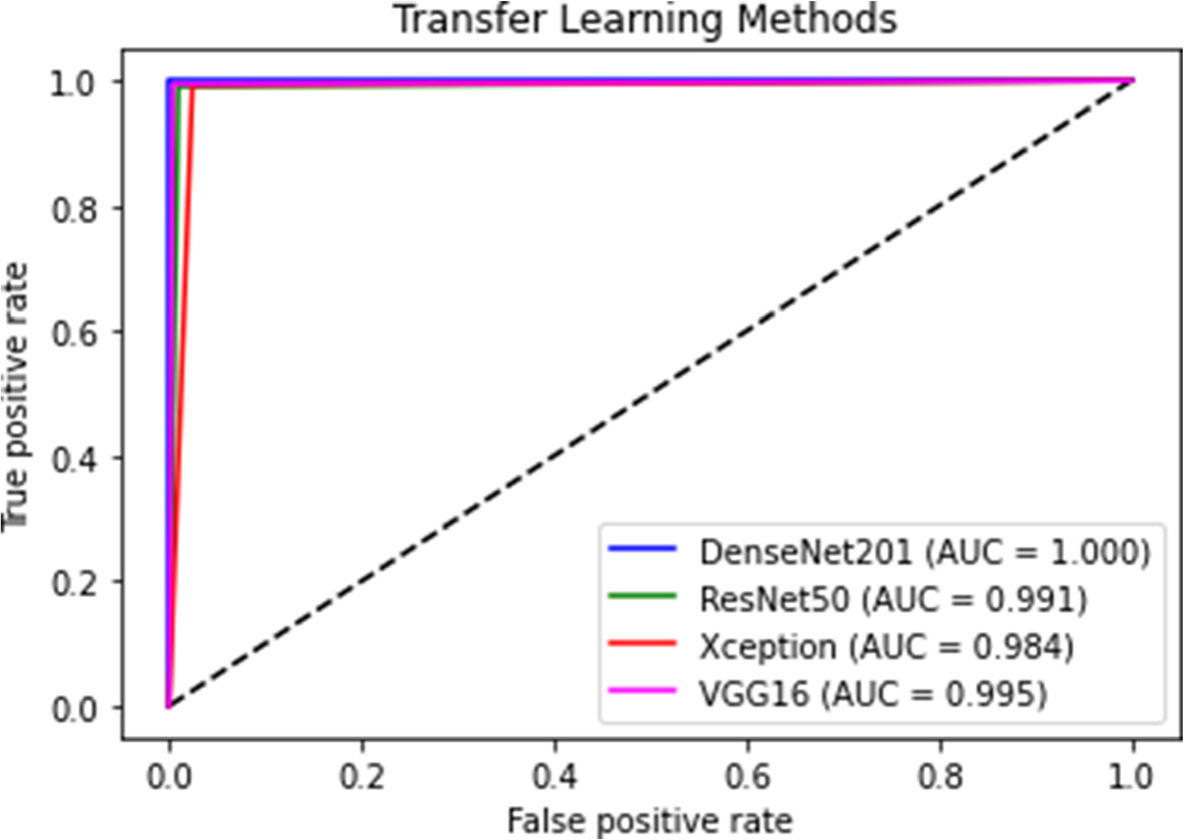
ROC plots of the pre-trained models

In Figs. 5 and 6, confusion matrix and receiver operating characteristic curve (ROC) plots of the models are given, respectively. With the help of the confusion matrix, the impact of FP and FN rates in models’ performance is shown. It clearly indicates that the DenseNet201 model provides not any FP and FN rates. However, the ResNet50, Xception, and VGG 16 models also classified 5, 4, and 3 cases, respectively, as FP. They belonged to the suspected COVID-19 group, but the models mistakenly placed them in the confirmed COVID-19 group. The ResNet50, Xception, and VGG 16 models classified 5, 13, and 2 cases, respectively, as FN. They belonged to the confirmed COVID-19 group, but the models mistakenly placed them in the suspected COVID-19 group. As a result, the DenseNet201 pre-trained model provides superiority over the other models in recognizing COVID-19-infected patients by chest CT images.

### An analysis of combining the pre-trained models with the ML methods

**Fig. 7:**
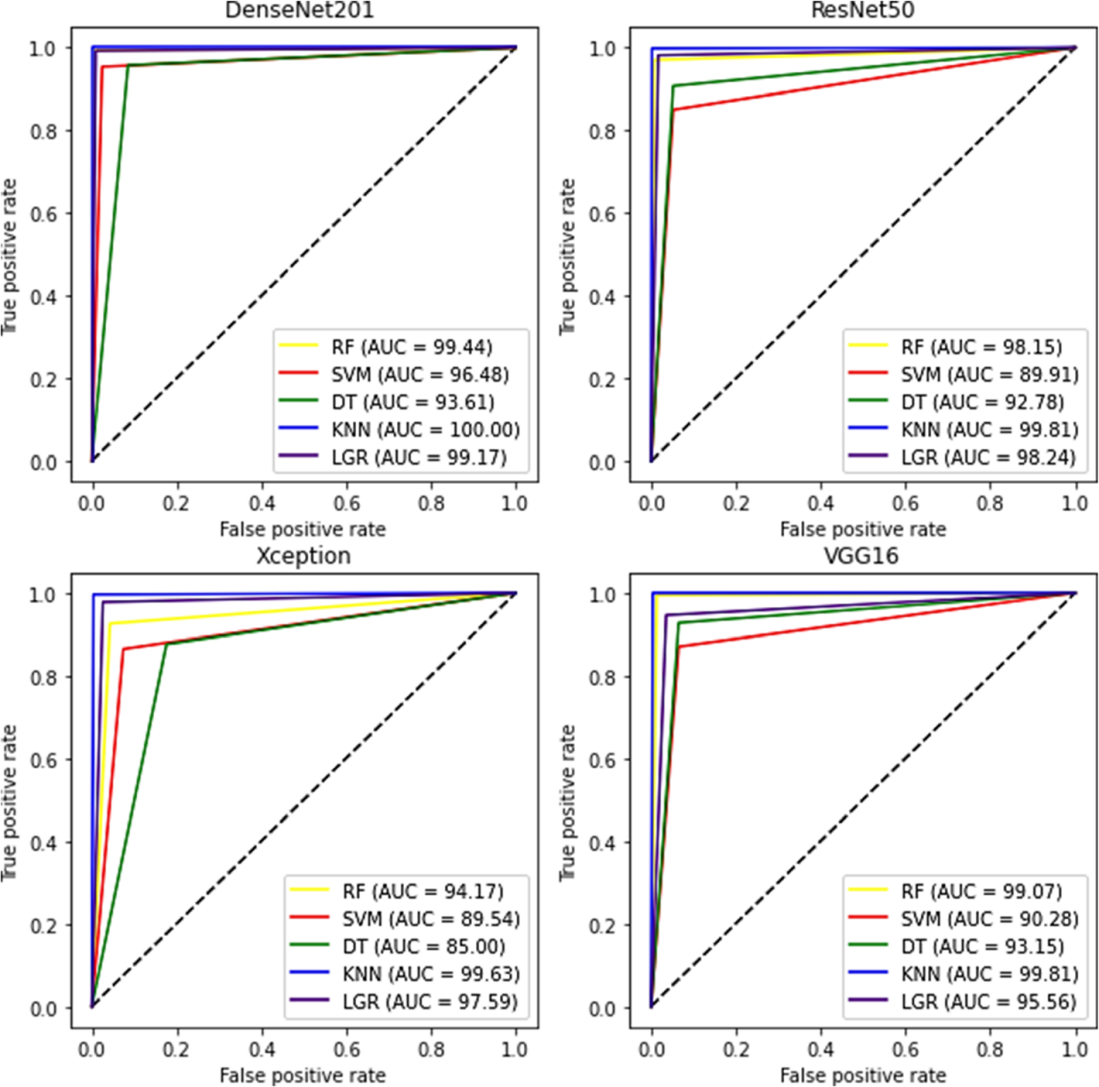
ROC plots of the pre-trained models in combination with five ML classifiers

The combination of pre-trained models with ML algorithms, including RF, SVM, DT, KNN, and LGR, are presented in Table 2 Similar to the pre-trained model results, the highest training performance metrics were obtained with the DenseNet201 model. The DenseNet201 model and KNN algorithm have received the best performance as the accuracy of 100%, recall of 100%, the precision of 100%, and f1-score of 100%. Results showed that the KNN classifier, in combination with pre-trained models, has a strong performance. As shown, ResNet50, Xception, and VGG16 models, combined with the KNN classifier, have almost the same and high performance compared to other classifiers. The lowest performance values were yielded as an accuracy of 85%, recall of 85%, precision of 85.10%, and f1-score of 84.98% for the Xception model and DT classifier. As a result, the KNN classifier for screening and diagnosing COVID-19 disease provides superiority over the other ML classifiers. Figure 7 depicts the AUC of the pre-trained models in combination with ML classifiers. The model DenseNet201+KNN classifier achieved the highest AUC (AUC, 100), followed by ResNet50 +KNN classifier and VGG16 +KNN classifier (AUC, 99.81).

**Table 2:** The results were obtained by applying pre-trained models in combination with five ML classifiers

Model	Classifier	Performance metrics (%)			
		Accuracy	Recall	Precision	F1 score
DenseNet201	RF	99.44	99.44	99.45	99.44
	SVM	96.48	96.48	96.50	96.50
	DT	93.61	93.61	93.69	93.60
	KNN	100	100	100	100
	LGR	99.16	99.16	99.17	99.16
ResNet50	RF	98.14	98.14	98.17	98.15
	SVM	89.9	89.9	90.29	89.88
	DT	92.77	92.77	92.84	92.77
	KNN	99.81	99.81	99.81	99.81
	LGR	98.24	98.24	98.24	98.24
Xception	RF	94.16	94.16	94.21	94.16
	SVM	89.53	89.53	89.68	89.52
	DT	85	85	85	84.98
	KNN	99.62	99.62	99.63	99.62
	LGR	97.59	97.59	97.59	97.59
VGG16	RF	99.07	99.07	99.08	99.08
	SVM	90.27	90.27	90.44	90.26
	DT	93.14	93.14	93.15	93.14
	KNN	99.81	99.81	99.81	99.81
	LGR	95.55	95.55	95.57	95.55

## Discussion

The new coronavirus (COVID-19) spread has been of great concern to the global community because it threatens the health of billions of humans, and there is no effective treatment to cure the disease. The early diagnosis of COVID-19 has been made possible with rapid and accurate image processing methods regarding the computation approaches. In this field, deep transfer learning models have a tremendous advantage in giving faster and better outcomes. Deep transfer learning techniques are widely used in the automatic analysis of medical images. These techniques can train the weights of networks on large datasets and fine-tuning the weights of these networks on small datasets. Due to the small COVID-19 dataset available, we used the DenseNet201, ResNet50, VGG16, and Xception models for fine-tuning these networks’ weights on the data set. Recent studies identified that ML algorithms could be applied to discover patients’ subgroups and for clinical decision guidance. In the current study, to classify patients, we used ML methods, including RF, SVM, DT, KNN, and LGR, in combination with pre-trained models. Nowadays, machine and deep learning techniques have developed as veritable methods to improve technologies across all domains and applications, including disease diagnosis and treatment. In this study, for helping the battle against COVID-19 disease, deep transfer learning models and ML algorithms were proposed to predict COVID-19 disease using chest CT images automatically.

Recently, advances in deep learning methods have played a significant role in the diagnosis of COVID-19 disease. Toğaçar et al. [42] used pre-trained CNN models, including AlexNet, VGG-16, and VGG-19, to determine pneumonia. The dimension of the features was reduced using the minimum redundancy maximum relevance (mRMR) algorithm. Then, the features obtained by the mRMR feature selection algorithm were combined, and this feature set was applied as the input to machine learning algorithms including, KNN, linear discriminant analysis (LDA), linear regression (LR), and SVM. Finally, the LDA with an accuracy of 99.41% yielded the most efficient results. We did not apply any feature extraction methods, and the models had an end-to-end architecture in comparison with Toğaçar et al. study. Also, we gained more accuracy in the combination of pre-trained models with ML algorithms. In this study, the accuracy of the VGG16 pre-trained model was in close agreement with the overall classification accuracy of Toğaçar et al.’s study. Das et al. [43] used the transfer learning model of Inception (Xception) to detect COVID-19. Their proposal consisted of convolution layers, max pooling, stride, global average pooling, and fully connected. They achieved a detection accuracy of 0.974 using chest X-ray images. We gained more accuracy for the Xception model in comparison with the Narayan Das et al. study. Moreover, we reached the accuracy of 99.62% for the Xception model combined with the KNN classifier. In another study, Toğaçar et al. [44] trained the three datasets (COVID-19, pneumonia, and normal chest images) using the MobileNetV2 and SqueezeNet deep learning models and then classified them using the SVM method. The overall classification accuracy was 99.27%. In our study, the overall classification accuracy for the DenseNet201 model in combination with the SVM classifier was in close agreement with Toğaçar et al.’s data. Nevertheless, we assessed several pre-trained models and ML algorithms and gained more accuracy than Toğaçar et al. study. The classification accuracy reached 100% for the pre-trained DenseNet201 model and DenseNet201+KNN classifier. Ozturk et al. [45] proposed a model for accurate diagnostics of binary classification and multi-class classification of COVID-19. Their model acquired a classification accuracy of 98.08% and 87.02% for binary classes and multi-class cases, respectively. In comparison with Ozturk et al. study, we assessed several models, and classification accuracy reached 100%. Song et al. [46] developed an accurate computer-aided procedure for helping clinicians in identifying COVID-19-infected patients by CT images. They collected chest CT images of 88, 101, and 89 patients diagnosed with the COVID-19, bacterial pneumonia, and healthy persons, respectively. The results showed that the proposed model could accurately identify the COVID-19 patients from the healthy with an AUC of 0.99, recall of 0.93, and precision of 0.96. However, we obtained a classification accuracy of 100%. Ismael et al. [47] used a deep-learning-based approach, fine-tuning of pretrained CNN, and end-to-end training of a developed CNN model to classify patients diagnosed with the COVID-19 and healthy persons using chest X-ray images. They used several pre-trained deep CNN models for deep feature extraction, including ResNet18, ResNet50, ResNet101, VGG16, and VGG19. For the classification of the deep features, the SVM classifier was used. The ResNet50 model and SVM classifier were obtained the highest accuracy score with 94.7% among all the obtained results. In our study, in contrast with Ismael et al., the classification accuracy reached 100% for the pre-trained DenseNet201 model and KNN classifier. Also, following used models in the Ismael et al. study, for the ResNet50 and the SVM classifier (DenseNet201+SVM classifier), we obtained accuracy 99.1 and 96.48, respectively. Zhou et al. [48] proposed an ensemble deep transfer learning model for COVID-19 detection in CT images. They have obtained 2933 lung CT images from COVID-19 patients. The average classification accuracy of the ensemble model was 99.05%. Of note, we gained more accuracy in the combination of pre-trained models with ML algorithms and pre-trained models.

In summary, the DenseNet201 and DenseNet201+KNN classifier models were promising for the diagnosis of COVID-19 based on the transfer learning and machine learning regarding achieved results and classification accuracy of 100% and can be used as an effective method for application in clinical routines. There were some limitations for this study which can be improved in future researches. We used the deep learning and ML algorithms with a dataset of chest CT images for COVID-19 positive cases along with suspected cases for training the models; however, other lung diseases such as lung opacity (Non-COVID lung infection) and viral pneumonia can be added to the database. This work can also be extended by adding risk and survival prediction of confirmed/suspected or other lung patients to help healthcare planning and management strategies.

## Conclusion

In the present study, for detecting and classifying COVID-19 disease from chest CT images, a deep transfer learning model and a deep transfer learning model combined with an ML classifier are proposed. These models are fully automated with an end-to-end structure, and there is no need to use the feature selection process, and they can perform classification with an accuracy of 100%. Therefore, the mentioned models can be used in remote places, in low- and middle-income countries, and laboratory equipment with limited resources to overcome a shortage of radiologists. A limited number of radiologists are present in every clinic to interpret CT images.

## Data Availability

The datasets used and/or analyzed during the current study are available from the corresponding author on reasonable request.

## Abbreviations

ML: Machine learning
SARS-CoV-2: Severe acute respiratory syndrome coronavirus 2
RT-PCR: Reverse transcription-polymerase chain reaction
LAMP: Loop-mediated isothermal amplification
LFAs: Lateral flow assays
ELISA: Enzyme-linked immunosorbent assay
CT: Computed tomography
SVMs: Support vector machines
RF: Random forest
DT: Decision tree
LGR: Logistic regression
KNN: K-nearest neighbors
CNNs: Convolutional neural networks
